# Feasibility and Preliminary Efficacy of a Community-Based Addiction Rehabilitation Electronic System in Substance Use Disorder: Pilot Randomized Controlled Trial

**DOI:** 10.2196/21087

**Published:** 2021-04-16

**Authors:** Xiaomin Xu, Shujuan Chen, Junning Chen, Zhikang Chen, Liming Fu, Dingchen Song, Min Zhao, Haifeng Jiang

**Affiliations:** 1 Shanghai Mental Health Center Shanghai Jiao Tong University School of Medicine Shanghai China; 2 Nantong Winner Information Technology Co Ltd Nantong China; 3 Council of Shanghai Ziqiang Social Services Shanghai China; 4 Council of Shanghai Zhongzhi Social Services Shanghai China; 5 Shanghai Key Laboratory of Psychotic Disorders Shanghai China; 6 Shanghai Clinical Research Center for Mental Health Shanghai China; 7 Center for Excellence in Brain Science and Intelligence Technology Chinese Academy of Sciences Shanghai China

**Keywords:** mobile health, drug use, rehabilitation, community health service, China

## Abstract

**Background:**

Drug use disorder has high potential for relapse and imposes an enormous burden on public health in China. Since the promulgation of the Anti-drug law in 2008, community-based rehabilitation has become the primary approach to treat drug addiction. However, multiple problems occurred in the implementation process, leading to a low detoxification rate in the community. Mobile health (mHealth) serves as a promising tool to improve the effectiveness and efficiency of community-based rehabilitation. Community-based addiction rehabilitation electronic system (CAREs) is an interactive system for drug users and their assigned social workers.

**Objective:**

The study aimed to examine the feasibility and preliminary efficacy of CAREs in community-based rehabilitation from the perspective of drug users and social workers in Shanghai, China.

**Methods:**

In this pilot randomized controlled trial, 40 participants were recruited from the community in Shanghai from January to May 2019. Participants randomized to the intervention group (n=20) received CAREs + community-based rehabilitation, while participants in the control group (n=20) received community-based rehabilitation only for 6 months. CAREs provided education, assessment, and SOS (support) functions for drug users. The assigned social workers provided service and monitored drug use behavior as usual except that the social workers in the intervention group could access the webpage end to obtain drug users’ information and fit their routine workflow into CAREs. The primary outcome was the feasibility of CAREs, reflected in the overall proportion and frequency of CAREs features used in both app and webpage end. The secondary outcomes were the effectiveness of CAREs, including the percentage of drug-positive samples, longest period of abstinence, contact times with social workers, and the change of Addiction Severity Index (ASI) from baseline to the 6-month follow-up.

**Results:**

The number of participants logged in to the app ranged from 7 to 20 per week, and CAREs had relatively high levels of continued patient use. Drug users preferred assessment and education features in the app end while their social workers showed high levels of use in urine results record and viewing assessment results on the webpage end. After the 6-month intervention, 3.3% (17/520) of samples in the intervention group and 7.5% (39/520) in the control group were drug-positive (F=4.358, *P*=.04). No significant differences were noted between the control and intervention groups in terms of longest duration of abstinence, number of contact times and ASI composite scores.

**Conclusions:**

The study preliminarily demonstrated that with relatively good feasibility and acceptability, CAREs may improve the effectiveness and efficiency of the community-based rehabilitation, which provided instruction for further improvement of the system.

**Trial Registration:**

ClinicalTrials.gov NCT03451344; https://clinicaltrials.gov/ct2/show/NCT03451344

**International Registered Report Identifier (IRRID):**

RR2-10.3389/fpsyt.2018.00556

## Introduction

In China, illicit drug abuse is an increasingly serious and complicated problem. An estimated 2.4 million people used illicit drugs by the end of 2018, imposing a significant burden on the addiction treatment system [1]. According to articles 33, 38, and 49 of the Anti-Drug Law, drug users are ordered to receive treatment for addiction in three forms: medical institution–based detoxification, community-based rehabilitation, and isolated compulsory treatment [2], among which the community-based rehabilitation is the primary approach for drug rehabilitation in China [3].

Community-based rehabilitation provides counseling and support, monitors drug use behavior to help drug users maintain long-term abstinence and promote their social integration. As the most economically developed city in China, Shanghai has more than 1000 social workers who serve as not only the main supervisor but also the helper for drug users, playing a significant role in the implementation of community-based rehabilitation [4]. However, due to scarcity of effective prevention and intervention within the community, drug users cannot mitigate risks associated with drug use, which may result in adverse consequences: relapse, incarceration, or death [5]. This dilemma arises in part from the immature development of community-based rehabilitation, which is reflected in inadequate facilities and funding sources in community or nonprofessional services and delayed feedback from social workers [6]. It is also a challenge for both social workers and drug users to maintain 3-year drug rehabilitation according to national legislation [2]. Therefore, it is essential to develop novel interventions to improve the effectiveness and efficiency of community-based rehabilitation, which can provide enormous benefit to public health.

Bringing mobile health (mHealth) into the routine treatment regimen may make it possible by delivering evidence-based health information, ongoing monitoring, and personalized intervention according to collected data on patients via sensors, apps, webpages, and location-tracking technology, which can improve treatment adherence, patient-provider communication, and recovery from diseases [7,8]. The past decade has seen the emergence of mHealth for chronic disease management including substance abuse [9], primarily in the means of short message service (SMS, or text messaging) or phone calls [10]. Compared with traditional technologies, smartphones show significant advantages for supporting complicated apps, accessing measurements with built-in mobile sensors, and allowing an omnipresent internet connection [11]. In China, smartphones are now widely used, with around 713 million users in 2018 [12]. Strategies for drug rehabilitation such as information or education, social support, assessment, feedback, monitoring, skills training, psychological intervention, self-management, and relaxation could be realized by smartphones app [13,14]. Although several apps for substance-related and addictive disorders have proved to be effective in randomized clinical trials, most of the apps are designed for alcohol and nicotine abuse [15,16]. Meanwhile, to the best of our knowledge, no app interventions with integrated functions for improving drug users’ antirelapse skills, increasing working efficiency, and enhancing interaction between social workers and drug users have been used in community-based rehabilitation.

Due to the current context in China, our research team developed a community-based addiction rehabilitation electronic system (CAREs) centered on a smartphone app with the aim of improving the professionalization and efficiency of the community-based rehabilitation [17]. This paper reports findings from a pilot randomized controlled trial demonstrating the feasibility and preliminary efficacy of CAREs in community-based rehabilitation.

## Methods

### Study Design

This study was a randomized controlled trial with 2 parallel groups comparing preliminary efficacy of CAREs + community-based rehabilitation to community-based rehabilitation alone. The protocol was registered at ClinicalTrials.gov [NCT03451344] and previously published [17]. The study was approved by the Shanghai Mental Health Center Ethics Committee (2017-33) and was in accordance with the principles of the Declaration of Helsinki.

### Participants

Participants newly designated to receive community-based rehabilitation were enrolled (January 2019 to May 2019) from the social worker station in Shanghai in this study. All participants were recruited through advertisements in the Ziqiang and Zhongzhi consortia, the 2 largest specialized social worker consortia in Shanghai to help drug users in the community. Inclusion criteria were aged 20 to 50 years, met the *Diagnostic and Statistical Manual of Mental Disorders, Fifth Edition* criteria for substance use disorders (SUD), and provided informed consent. Exclusion criteria were inability or refusal to use smartphone app, severe cognitive impairment, or a history of suicidality.

### Procedures

After signing the written informed consent, participants were screened for eligibility within 3 days. At this time, the demographic characteristics, drug use information, and urine drug screen (UDS) were collected by trained social workers. Participants who met the inclusion criteria received a 7-day training with their assigned social workers on how to use CAREs to familiarize them uniformly with it. Participants were randomly assigned to receive either CAREs + community-based rehabilitation or community-based rehabilitation only (1:1 ratio) for 6 months using simple randomization tables generated by SPSS Statistics version 22 (IBM Corp).

Each participant was assessed for severity of problems associated with drugs in 7 domains (alcohol use, drug use, medical, employment, legal, family/social, and psychiatric status) using the Chinese version of the Addiction Severity Index (ASI) at baseline and after the 6-month intervention. The Chinese version of the ASI has been proven with good reliability and validity [18-20]. UDS was collected once a week during the study course (26 weeks in total); samples were screened for heroin, amphetamine-type stimulants, marijuana, cocaine, and ketamine.

Social workers conducted assessments and weekly UDS in the social worker station of each subdistrict. The people who provided the CAREs intervention were different from those who performed UDS and assessment of ASI. However, it was not possible to completely blind the evaluators to group allocation as the participants in intervention group may talk about the CAREs intervention during the assessment. Participants in the intervention group were compensated with 50 RMB (US $7.64) for potential cost for mobile data, and at the end of the study they received a smartphone with the CAREs app installed.

### Intervention

#### Control Group: Community-Based Rehabilitation

According to national legislation [2], drug users who received regular community-based rehabilitation must sign an agreement to comply when they were newly enrolled in this program. They submitted a written report if they left their city of residence, in accordance with the localized management. Participants visited their assigned social worker and agreed to be tested for illicit drugs every 2 months. In this study, participants in the control and intervention groups were asked to submit a urine sample once a week. As those whose urine test result was positive would be sent to an isolated compulsory treatment center for 2 years, it was conservatively estimated that participants who did not or refused to submit urine samples were considered to have positive results on UDS. At other times, social workers helped their clients apply for social benefits as needed and provided counseling irregularly if necessary.

#### Intervention Group: CAREs + Community-Based Rehabilitation

The intervention group received the same community-based rehabilitation as the control group. In addition, they received access to CAREs, which consists of a smartphone app for drug users and a webpage for social workers. Participants were required to log in to the app at least once a week and encouraged to use it at other times. The app was designed with 3 major modules for drug users providing education, assessment, and coping skills and support.

The educational content was selected from a course on saying no to drugs and delivered in the form of text or video with material covering basic knowledge about drugs, confidence building, treatment principles, antidrug skills, and emotion management using cognitive behavioral therapy based on the relapse prevention model [21]. More specifically, clinical guidelines for stimulant-induced mental or somatic symptoms, replacement therapy, methadone maintenance treatment, the role of personal relationships in addiction treatment, and so on were introduced in the treatment principles component, and antidrug skills contained an introduction of self-help groups to create a support network, develop clear thinking about major life events and stress, resist temptation from all sides, cope with cravings, etc. These educational resources were delivered in a specific order once a week. Users were expected to complete the educational courses on time when receiving the reminder. To reinforce learning and withdrawal motivation, participants were encouraged to revisit lessons at any time and could earn points by viewing educational content and finishing the corresponding exercises after learning.

Assessments were provided using 5 self-report instruments with proven reliability in substance-using samples. Craving was assessed by visual analog scale [22], with 0 cm being no craving at all and 10 cm suggesting the most craving ever experienced. Scores of the Patient Health Questionnaire–9 [23] indicated the level of depression severity: minimal (0-4), mild (5-9), moderate (10-14), moderately severe (15-19), or severe (20-27). Scores of the General Anxiety Disorder–7 [24] indicated the level of anxiety severity: minimal (0-4), mild (5-9), moderate (10-14), or severe (15-21). The Alcohol Use Disorders Identification Test [25] was used to screen at-risk drinking: low-risk alcohol use (0-7), hazardous alcohol use (8-15), harmful alcohol use (16-19), or alcohol dependence (≥20). The family and employment status was adapted from the Chinese version of the ASI [18-20] and contained 6 yes/no questions, with 0-3 points indicating a nonideal family or employment status. Users received scores to indicate level of severity as well as the real-time feedback accordingly. If the scores were above the normal cutoff point, users were advised to learn the coping skills from the education and SOS module or turn to their assigned social workers. Meanwhile, the social workers would receive reminders to pay more attention to those users and initiate safety protocols when necessary. Participants were required to complete the assessment once a week.

The SOS module contained tools for skills coping, relaxation, and call forwarding to connect with contacts, including family members, doctors, and social workers. Skills coping with craving are vital due to the close connection between craving and relapse [26]. Relaxation training included music relaxation and abdominal breathing. Drug users could also interact with their assigned social workers on the message board through the app.

Meanwhile, social workers could obtain all information in CAREs apps of their assigned drug users through the webpage end. By using the webpage, social workers could record urine test results, track real-time location, review results of assessments, reply to messages, and send reminders if participants missed a urine test or assessment. If drug users left the supervision area in Shanghai without notifying the social worker, the system automatically initiated alarm but only the matched social worker received the reminder. Of note, all data would be stored in the server for safety and privacy considerations.

### Outcome

The primary outcome was feasibility of CAREs in the community-based rehabilitation program, reflected in the use of CAREs in both app and webpage ends, reported as the overall proportion and frequency of CAREs features used. Use was defined as a participant or social worker accessing a feature page (not the home page) in CAREs. However, data on mean count per user was not possible for CAREs due to technical reasons on the database end.

Secondary outcomes were effectiveness of CAREs, including (1) UDS results examined in overall percentage of drug-positive samples, (2) longest duration of sustained abstinence, defined as the greatest number of consecutive weeks of negative UDS samples in the 6-month period, (3) ASI composite scores summed according to the answers to each of the problem areas, and (4) contact times, days when participants interacted with their assigned social workers in the form of sending messages via CAREs, SMS, face-to-face meeting, or phone call.

### Statistical Analysis

Intention-to-treat principle was used in all analyses. The statistical analyses were conducted with SPSS Statistics version 22. Significance level was set at *P*<.05. Descriptive statistics were used to describe baseline data and CAREs feature use. Chi-square test for categorical variables and Student *t* test for continuous variables were used to examine the baseline comparability of the two groups. Percentage of drug-positive samples, longest period of abstinence, and contact times were examined for significant difference by analyses of variance. As for the ASI composite scores, repeated measure analysis of variance was used to evaluate the differences between baseline and 6-month scores. G*power program [27] was used for power analysis.

## Results

### Participant Characteristics

**Figure 1:**
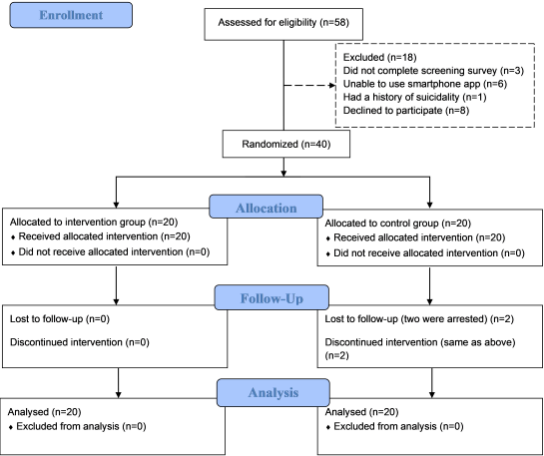
CONSORT flowchart of the study.

Figure 1 shows the participant flow. A total of 40 people were randomized into the intervention (n=20) and control (n=20) group of the trial, with only 2 participants (5%) in the control group dropping out due to being arrested (caught using illicit drugs by the police at weeks 20 and 25, respectively). Baseline demographic characteristics were presented in Table 1. No significant differences between the groups were found (*P*>.05).

**Table 1:** Baseline and demographic characteristics of participants (n=40).

Characteristic		Intervention (n=20)		Control (n=20)		t/χ^2^		*P* value
Age in years, mean (SD)		47.0 (8.8)		45.1 (11.0)		0.604		.55
Male, n (%)		16 (80)		15 (75)		0.143		.71
Employed, n (%)		9 (45)		6 (30)		0.960		.33
**Marital status, n (%)**		—^a^		—		0.895		.24
	Married	6 (30)	11 (55)		—		—	
	Divorced	8 (40)	4 (20)		—		—	
	Never	6 (30)	5 (25)		—		—	
**Education, n (%)**		—		—		1.423		.49
	Low (<6 years)	0 (0)	1 (5)		—		—	
	Middle (6-9 years)	14 (70)	13 (65)		—		—	
	High (>9 years)	6 (30)	6 (30)		—		—	
Accumulated years of drug use, mean (SD)		10.7 (7.9)		10.1 (7.2)		0.272		.79
**Type of primary drug use, n (%)**		—		—		0.360		.55
	Methamphetamine	19 (95)	18 (90)		—		—	
	Heroin	1 (5)	2 (10)		—		—	

^a^Not applicable.

### Use of CAREs

#### Features on Webpage End for Social Workers

**Figure 2:**
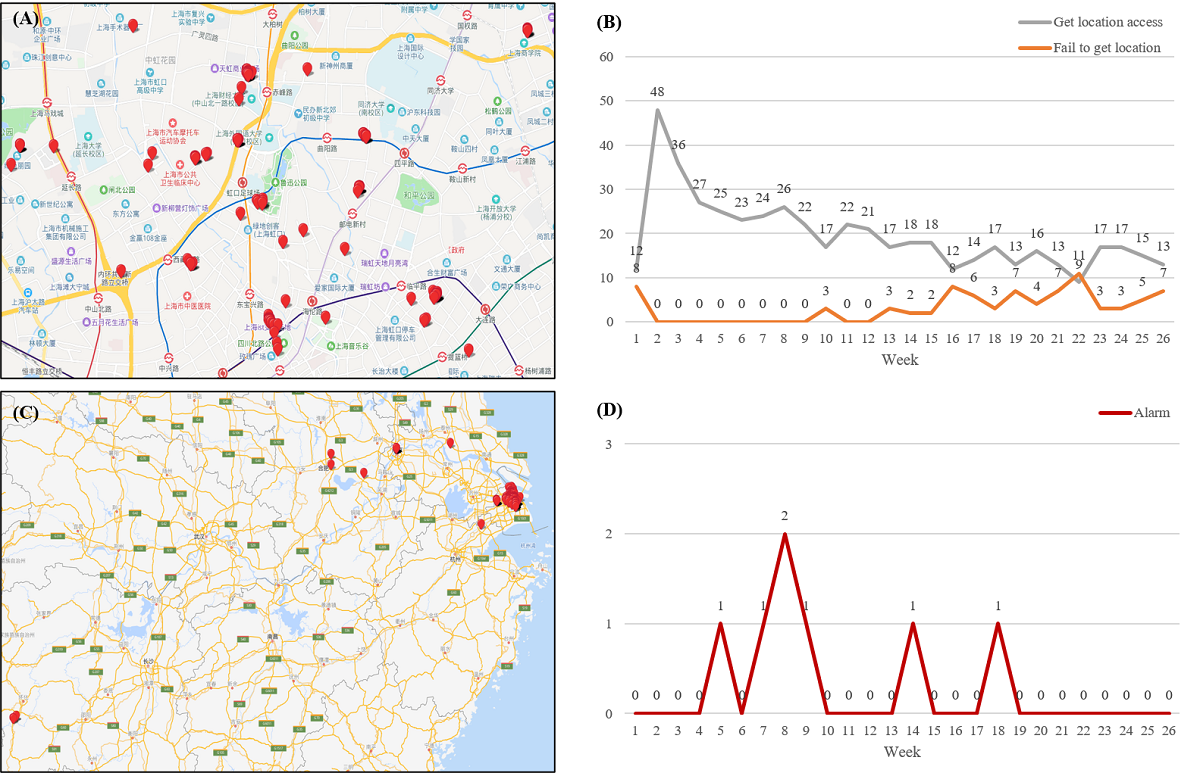
Location-tracking feature for social workers to monitor participants in intervention group: (A) close shot of Shanghai: drug users moving around within the supervision area; (B) number of locations accessed and not accessed (failed) from CAREs app per week; (C) remote view of Shanghai: some drug users had left the supervision area without reporting to the matched social workers, and the system automatically initiated alarm; and (D) number of alarms per week.

Uses of CAREs features on both webpage and app ends were shown in Table 2. For social workers, the 3 most commonly used functions were recording urine test results, location tracking, and viewing assessment information of drug users, with 100% (20/20), 80% (16/20), and 60% (12/20), respectively, of social workers using them on at least one occasion. Reminders for urine test and assessment were the least used. However, when it came to mean number of times over the whole study course, use of these features was relatively low. Over the study period, social workers accessed urine tests record and assessment results an average of 32.87 times and 26.56 times, respectively. Location tracking was the third most frequently used, and data from the back-end showed that the number of times accessing location of participants in the intervention group decreased over time, while the system failed to get some locations in the latter half of the study (Figure 2A and B). When the drug users left the supervision area in Shanghai without submitting a written report, the system automatically initiated alarm 7 times in total (Figure 2C and D).

**Table 2:** Use of CAREs features on both webpage and app ends.

Features			Rate of users with at least one use (%)	Mean total^a^
**Social workers (webpage end)**				
	Record urine test results		20 (100)	32.87
	Location tracking		16 (80)	12.04
	View assessment information		12 (60)	26.56
	Send urine test reminder		7 (35)	3.47
	Send assessment reminder		3 (15)	0.65
**Drug users (app end)**				
	Assessment		20 (100)	24.15
	Education		15 (75)	63.3
	**SOS**			
		Music relaxation	9 (45)	2.65
		Abdominal breathing	7 (35)	1.6
		Hotline for doctors	3 (15)	0.25
		Hotline for voluntary drug rehabilitation center	3 (15)	0.25
		Call family number	9 (45)	1.75

^a^Mean total: mean number of times across the participants in the intervention group over the whole study course.

#### Features on App End for Drug Users

**Figure 3:**
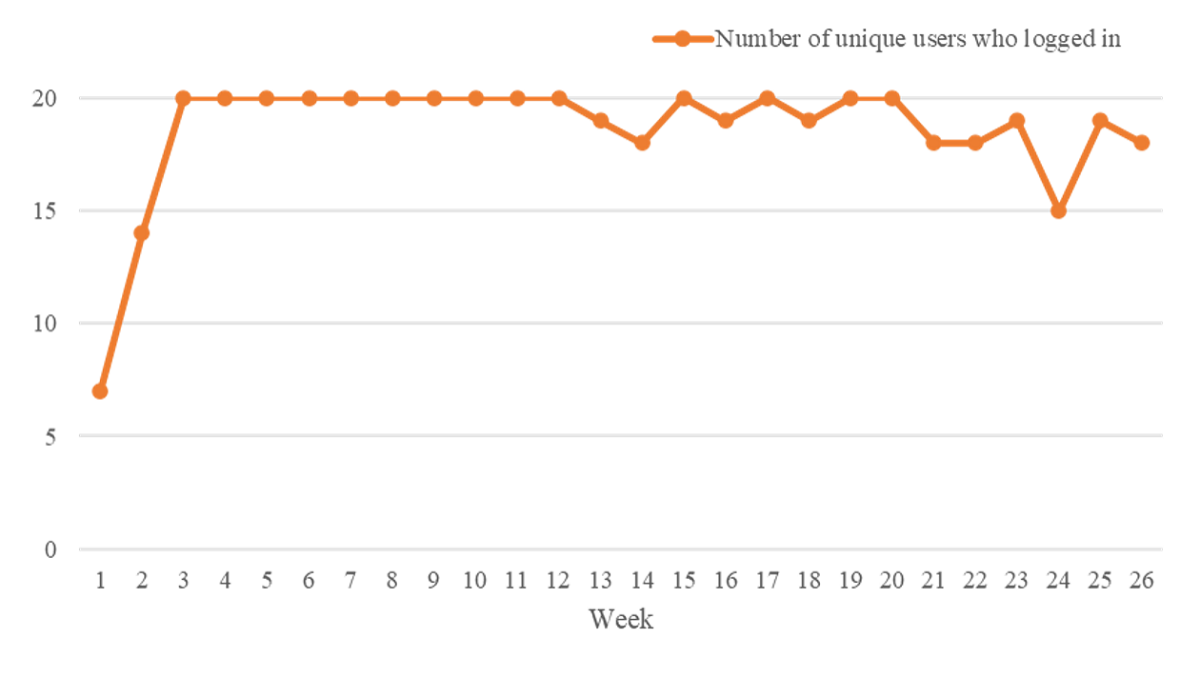
Number of unique participants who log in to the CAREs app at least once each week.

**Figure 4:**
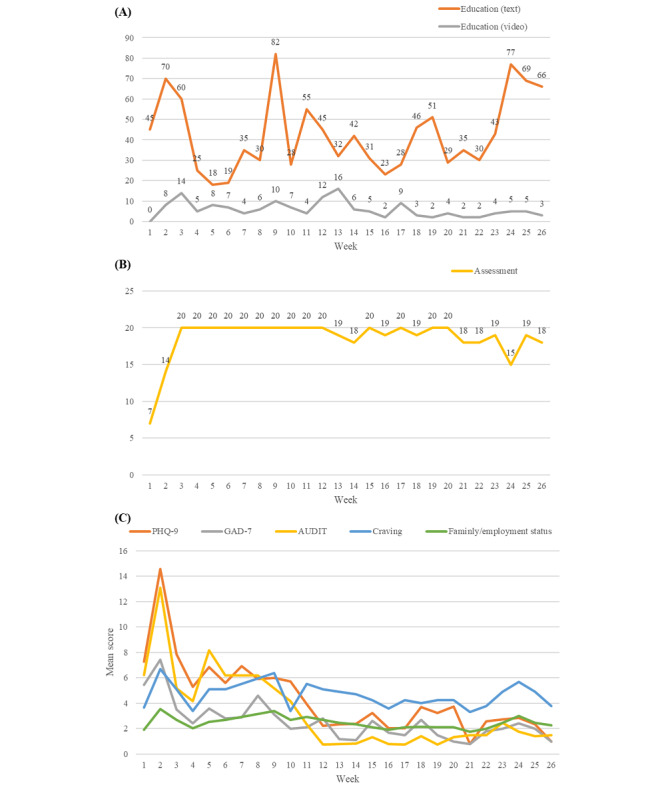
(A) Total number of people using education (in the form of text or video) function from CAREs app per week; (B) number of unique users who used assessment function from CAREs app per week (maximum number of people was 20); (C) mean scores of assessment results decreased over time.

The number of participants logged in to the app ranged from 7 to 20 per week, and CAREs had high levels of continued patient use, as shown in Figure 3. In terms of content analysis of CAREs app for drug users (Table 2), the assessment feature was accessed by the largest number of users (20/20, 100%), followed by education (15/20, 75%). However, the greatest average number of uses over the 6-month period was education, which was shown in Figure 4A. Educational content delivered by text message was preferred by participants over video. Number of unique users who used the assessment function of the CAREs app per week is shown in Figure 4B, and Figure 4C displays the assessment results per week. At the beginning of the study, especially in the second week, mean scores of assessments indicated that participants were generally in the moderately severe range of depression, mild anxiety, and hazardous drinking. These mean scores decreased over time and maintained a low level in the last few weeks. However, drug users maintained nonideal family and employment status and the same level of craving during the 6-month period.

**Figure 5:**
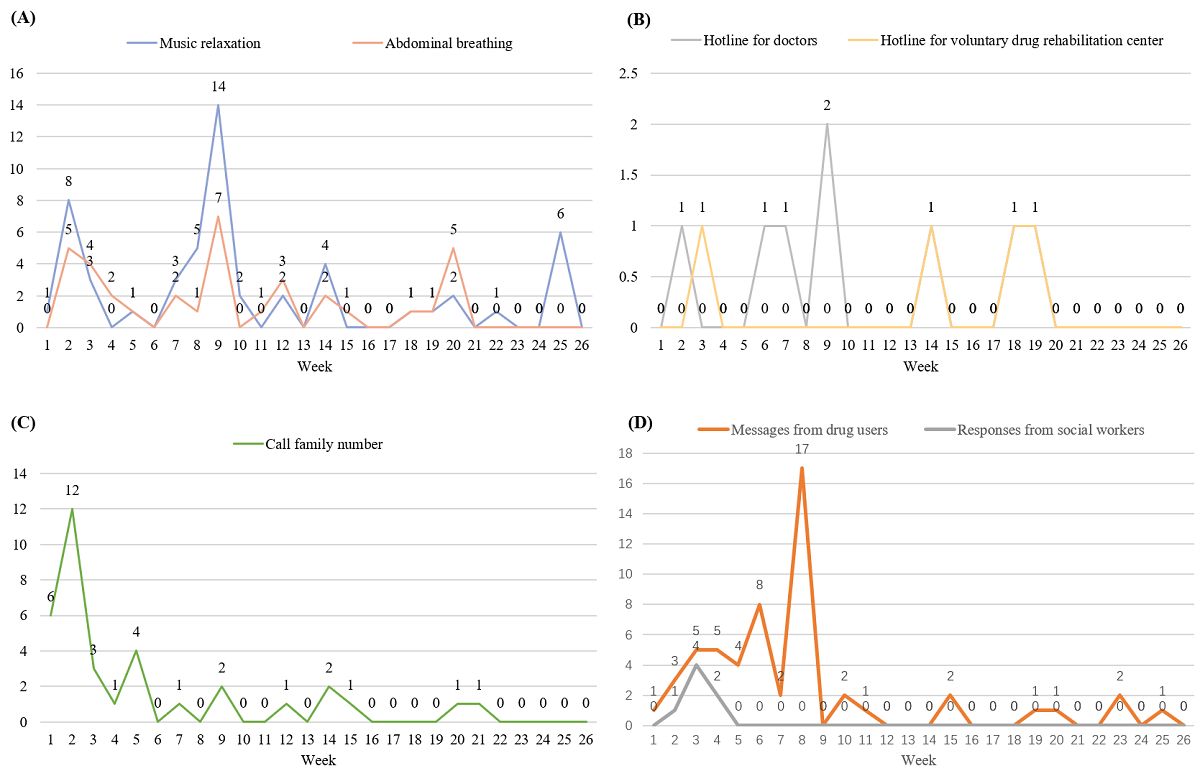
Mean number of times of SOS features per week: (A) relaxation training (including music relaxation and abdominal breathing); (B) call forwarding service to doctors and voluntary drug rehabilitation center; (C) call forwarding service to family members; (D) message board used in both drug user and social worker ends.

Data on SOS functions showed low levels of use (Table 2), with a relatively small proportion of participants choosing music relaxation or abdominal breathing to cope with craving and emergency. Figure 5A to 5C showed that the frequency of use of some SOS features (such as calling family members) peaked at first but decreased over time, while the hotline for doctors and voluntary drug rehabilitation center maintained low frequency of use all the time. As for the message board shown in Figure 5D, drug users left messages frequently in the first few weeks but decreased at seventh week, with low responses from social workers all the time.

### Drug-Related Outcomes and Contact Times

The follow-up assessment was conducted 0 days to 5 days postintervention (mean 0.4), and there was no difference between groups (*t*=11.427, *P*=.06). Descriptive and statistical tests are shown in Table 3. There were 987 urine samples collected in total during the 6-month study period (weeks 20 to 26; mean 24.7). Participants in the intervention group showed a lower percentage of drug-positive samples than participants in the control group. No significant differences were found in terms of longest period of abstinence, ASI composite scores, or contact times. The power to compare percentage of drug-positive samples between the 2 groups was 0.74, with longest period of abstinence 0.73, contact times 0.16, and ASI composite scores ranging from 0.07 to 0.75.

**Table 3:** Effectiveness of outcomes: drug use and contact times.

Variables		Intervention (n=20)		Control (n=20)		F score	*P* value
		Baseline	End of 6 months	Baseline	End of 6 months		
% drug-positive samples, mean (SD)		—^a^	3.3 (5.0)	—	7.5 (7.5)	4.358	.04
Longest period of abstinence, mean (SD)		—	24.65 (2.21)	—	22.80 (3.59)	3.851	.06
Contact times, mean (SD)		—	42.45 (18.51)	—	36.65 (17.48)	1.038	.32
**ASI^b^ composite scores, mean (SD)**							
	Medical status	0.21 (0.27)	0.07 (0.29)	0.15 (0.26)	0.04 (0.13)	0.316^c^	.58
	Employment status	0.74 (0.28)	0.07 (0.22)	0.67 (0.31)	0.05 (0.23)	0.636^c^	.43
	Alcohol use	0.05 (0.80)	0.01 (0.03)	0.13 (0.16)	0.02 (0.15)	1.672^c^	.20
	Drug use	0.05 (0.07)	0.01 (0.03)	0.04 (0.04)	0.01 (0.03)	0.302^c^	.59
	Legal status	0.04 (0.08)	0.00 (0.11)	0.05 (0.13)	0.02 (0.08)	0.025^c^	.87
	Family/social status	0.10 (0.13)	0.03 (0.10)	0.10 (0.13)	0.04 (0.11)	0.011^c^	.92
	Psychiatric status	0.06 (0.06)	0.03 (0.06)	0.05 (0.07)	0.03 (0.07)	0.043^c^	.84

^a^Not applicable.

^b^ASI: Addiction Severity Index.

^c^Statistical tests represent effects of group × time interaction.

## Discussion

### Principal Findings

To our knowledge, this was the first pilot study examining the feasibility and preliminary efficacy of a smartphone app (CAREs) to facilitate community-based rehabilitation program from the perspective of the drug users and social workers in China. The findings of this study provided preliminary evidence of CAREs as a potential tool with moderately good acceptability and effectiveness among individuals mainly using methamphetamine from community-based rehabilitation program. This was reflected in better performance on the UDS in the intervention group and moderate to high engagement with some CAREs features. Unexpectedly, the previous hypothesis that CAREs intervention would significantly prolong longest period of sustained abstinence, increase contact times, and reduce ASI composite scores was not confirmed in this study.

Growing awareness of the impact of addiction on public health calls for broader reach with lower barriers to services for drug users [28,29]. However, traditional support services may bring drug users concerns about relevance and stigma, creating barriers to access to routine treatment [30,31]. Under the addiction treatment model and system in contemporary China, studies examining the feasibility of mHealth among drug users have mainly been conducted in patients using heroin from methadone maintenance treatment (MMT) programs. Han [32] found poor acceptance of a mobile phone–based ecological momentary assessment app in the MMT population. Liang [4], however, demonstrated the feasibility and potential benefits for participants in the MMT program to receive both surveys and text messages from the S-Health app. As there is still a large deficiency in social workers’ time and professional competence to provide evidence-based interventions in community-based rehabilitation program [33], the significance of the role of social workers was also considered in this study. CAREs is an interactive system for drug users and their matched social workers to address problems of addiction.

Like other mHealth app interventions [34,35], although nearly all participants logged in to CAREs during each week in the 6-month period, a gradual decline of app engagement was found in this study. Some flaws of CAREs may hinder operations. For example, some clients could not log in to their accounts or view repeated content because of technical difficulties. Nevertheless, the functions of self-monitoring, education, urine results records, and viewing assessment results showed relatively high use. As shown in this study, long-term education and gradually improved assessment results may contribute to the lower percentage of drug-positive samples in the intervention group. Some participants in the intervention group mentioned that they felt proud when the assessment results improved and became more confident to overcome the addiction by knowing more about drug-related knowledge. According to the social cognitive theory [36,37], self-monitoring such as the assessment and education features of the CAREs app would increase self-efficacy beliefs which operate with goals, positive outcome expectancies, and environmental perception to facilitate one’s motivation and behaviors. In line with previous studies of SUD intervention, repeated assessments improved self-monitoring [38,39]. Education increased awareness of potential risks [40-42] and skills to prevent relapse [43,44] and thereby helped individuals change their dysfunctional behaviors.

On the other hand, compared with the existing routine face-to-face interviews, the help and education of social workers was more convenient through the trial implementation of CAREs, especially in terms of real-time feedback. Social workers requested that the CAREs app data be integrated into their existing management system, which could fit into their existing workflow, improve their work efficiency, and help them be more familiar with drug users’ situations. Some social workers mentioned that negative affect of drug users in the intervention group was detected earlier and more easily through the assessment information, and they would pay more attention to that drug user accordingly.

Although use of the location tracking with automatic alarm function was moderate, it indeed provided an effective method for better supervision of drug users within the city of residence. However, some drug users turned these features off with privacy concerns.

Additionally, use of SOS functions was relatively high initially but dropped to a low level of use in the later of the study course. Users may have accessed the features at first only because of freshness. The reason for low use of the call forwarding service and message board probably was that individuals with SUD preferred face-to-face interviews, which provided a way for them to communicate with others [32]. Similar finding has been reported in a feature analysis of a smartphone-based smoking cessation app: few participants used the Phone a Counselor function, as weekly offline counseling sessions made the hotline unnecessary or redundant [45]. Therefore, contact times between drug users and their assigned social workers in the intervention group was more than that in the control group, but not significantly.

In terms of reduced ASI composite scores, this was probably because ASI covers several domains associated with drugs, and CAREs did not provide components for legal and employment domains. This suggests that future iterations of the CAREs app should integrate more comprehensive components with the help of multicollaborations such as specialty addiction treatment settings, communities, and related administrative departments.

### Limitations

This study had several limitations. First, it should be acknowledged that this study was conducted only in Shanghai, where social workers were provided who specialized in helping drug users in the community due to legal requirements; furthermore, CAREs was designed with social workers as service providers. Therefore, considering different antidrug systems and procedures in other regions of China, the results of this study are for reference only. Second, the study terminated with only 40 samples because of the emergence of COVID-19, and the majority of the subjects were males. Although this study is promising, generalizability is limited by the small sample sizes and gender difference. Third, the mean count per user data could not be obtained from the database for technical reasons. As a pilot study, the relatively small sample size limited statistical power to detect the efficacy of CAREs, so the paper predominantly focused on the app’s feasibility. Technical support for the CAREs database should be improved to conduct secondary analysis of improved behavioral outcomes associated with the use of CAREs with larger samples. Also, technical problems may have prevented some users from engaging with the app. The related technical problems should be recorded in a future research process to distinguish whether problematic engagement is because of unwillingness or inability to use. Fourth, data on previous drug-related history (eg, UDS, period of abstinence, and treatment patterns) and information associated with comorbid symptoms were not collected in this study. More information should be requested in future studies to exclude the potential bias. Fifth, as addiction is a chronic disease [46], follow-up data are essential to evaluate whether the treatment is effective in maintaining abstinent after a 6-month intervention.

### Conclusions

This pilot study suggests a moderate level of feasibility and acceptability for CAREs in a community-based rehabilitation program. It preliminarily demonstrated that the support offered by CAREs may improve the effectiveness and efficiency of community-based rehabilitation. Future studies will focus on updating CAREs and conducting long-term effectiveness trials in well-powered and larger samples to improve the quality of rehabilitation for drug addiction in China.

## Appendix

Multimedia Appendix 1 CONSORT-eHEALTH checklist (V 1.6.1).

## Abbreviations

ASI: Addiction Severity Index
CAREs: community-based addiction rehabilitation electronic system
mHealth: mobile health
MMT: methadone maintenance treatment
SMS: short message service
SUD: substance use disorders
UDS: urine drug screen
